# An experimental study on the susceptibility of purchasing managers to greenwashing

**DOI:** 10.1038/s41598-025-94482-4

**Published:** 2025-04-03

**Authors:** Owais Khan, Andreas Hinterhuber

**Affiliations:** 1 Venice School of Management, Ca’ Foscari University of Venice, San Giobbe, Cannaregio 873, 30121 Venice, Italy; 2 School of Business, Aalto University, Ekonominaukio 1, 02150 Espoo, Finland

**Keywords:** Certifications, Cognitive biases, Greenwashing, Organizational procurement, Sustainability, Willingness to pay, Environmental social sciences, Sustainability

## Abstract

Greenwashing—the deliberate exaggeration or fabrication of environmental claims—undermines trust, disrupts transparency, and poses a significant barrier to genuine progress toward sustainability. This scenario-based experimental study examines whether purchasing managers, key stakeholders in organizational procurement, can reliably differentiate between greenwashed and certified sustainable products. Using three carefully designed purchasing scenarios—laptops, safety gloves, and copy paper—responses were collected from 465 purchasing managers across the EU, a region notable for its regulatory emphasis on eco-certifications. The findings reveal no statistically significant differences in willingness to pay (WTP) for products with greenwashed claims versus those backed by stringent certifications, with average WTP values varying only slightly between groups. These findings highlight a critical vulnerability to greenwashing, even among experienced professionals, raising concerns about the credibility of sustainability claims in influencing procurement decisions. The study underscores the need for systemic reforms, including the standardization of certification systems and enhanced decision-making tools, to mitigate greenwashing’s pervasive impact and foster authentic corporate sustainability.

## Introduction

Greenwashing—the practice of exaggerating or fabricating environmental claims—has become a significant barrier to achieving sustainability. Since its emergence in the 1980s, greenwashing has evolved in complexity, making it increasingly challenging for stakeholders to differentiate between authentic eco-friendly initiatives and deceptive marketing^1,2^. This practice not only erodes public trust but also distorts market competition, undermining collective efforts toward environmental goals^3–5^. Moreover, greenwashing scandals at the supply chain level have exposed the reputational and financial risks faced by organizations, often disrupting entire industries^6,7^. Despite its prevalence, research on greenwashing has primarily focused on consumers^8,9^, leaving a critical gap in understanding how organizational decision-makers, such as purchasing managers, navigate these challenges^10^.

Addressing greenwashing is vital for advancing sustainable development and fostering a circular economy^11^. The circular economy model, which emphasizes waste minimization and resource efficiency, relies on transparency and accountability across supply chains^12^. However, the increasing prevalence of greenwashing disrupts this transition by misleading stakeholders and promoting unsustainable consumption patterns^13–15^. Certifications have been developed as tools to combat greenwashing, offering verifiable claims to reassure stakeholders^16^. Yet, their effectiveness is undermined by the sheer number of eco-labels—exceeding 400 globally—and the lack of standardization across certification systems^4^. These challenges make it difficult for consumers—and likely even for experienced professionals, such as purchasing managers—to distinguish credible certifications from misleading claims^17,18^.

Purchasing managers play a pivotal role in advancing corporate sustainability, as their decisions influence supply chains, vendor relationships, and an organization’s overall environmental footprint^19^. However, their frequent exposure to diverse sustainability claims, combined with the complexity of certifications, increases their vulnerability to greenwashing. Despite their expertise, they may lack the necessary tools or frameworks to critically evaluate certifications, especially when faced with unstandardized or overlapping eco-labels. This vulnerability poses significant risks, as purchasing managers who fail to identify credible claims may inadvertently support unsustainable suppliers, undermining corporate sustainability goals and contributing to industry-wide credibility issues^4^.

While certifications have been shown to increase consumer willingness to pay (WTP) by approximately 7% on average^20^, it is reasonable to expect that purchasing managers, given their professional expertise, would exhibit an even greater WTP for certified sustainable products. Certifications not only validate sustainability claims but also signal trustworthiness, potentially enhancing perceived product value^21^. If, however, purchasing managers cannot distinguish between greenwashed claims and verified certifications, this represents a critical vulnerability to greenwashing, dramatically undermining the intended benefits of certifications in organizational contexts.

This study tests whether purchasing managers demonstrate a higher WTP for certified products compared to products with greenwashed claims. The central hypothesis is that the average WTP of purchasing managers should be higher for certified sustainable products. If this expectation is not met—meaning purchasing managers exhibit a similar or even greater willingness to pay (WTP) for greenwashed products—it would reveal that even trained professionals are vulnerable to greenwashing, posing a significant threat to the integrity of sustainable procurement practices.

The significance of this research is amplified by the increasing pressure on organizations to achieve ambitious environmental targets^22,23^. As purchasing managers navigate complex supply chains and an overwhelming array of eco-labels, their ability to make informed decisions becomes essential for aligning procurement practices with corporate social responsibility (CSR) and sustainability goals. This study not only contributes to academic knowledge by addressing a critical research gap but also offers practical insights to inform policy reforms, improve certification systems, and enhance decision-making support for purchasing and supply chain professionals^6,10^.

In summary, this study highlights the risks greenwashing poses to organizational procurement and the broader implications for sustainability. By shedding light on purchasing managers’ vulnerability to deceptive green claims, it underscores the need for transparent, standardized certifications and robust decision-making tools to foster genuine progress toward a circular economy. Insights from this research aim to strengthen sustainable procurement practices, mitigate greenwashing risks, and create a more reliable marketplace for environmentally responsible products.

## Methodology

### Study design

This study aimed to assess the vulnerability of purchasing managers to greenwashing—where misleading environmental claims might be perceived as favorably as genuine certifications. To achieve this, an experimental research design—involving three procurement scenarios—was employed. Scenario-based experiments have been widely used in purchasing and supply chain research as they are well-suited for examining decision-making processes in realistic yet controlled contexts^24,25^.

For this study, three product categories were selected: laptop, safety gloves, and copy paper. These products were chosen to represent common procurement scenarios across a wide range of industries. To ensure the relevance of these choices, two highly experienced purchasing professionals were consulted during the design phase.

The study framework was designed to assess differences in WTP by exposing one group of purchasing managers to greenwashed products (with increasing levels of complexity) and the other group to sustainable products (with increasing levels of certification credibility). This structured variation was a deliberate choice to enhance the experimental rigor and ensure a meaningful comparison of decision-making patterns (see Table 1).

**Table 1:** Study framework.

S. No	Product (Scenario)	Level of complexity (for Group A)	Level of credibility (for Group B)
1	Laptop	Mild Green Claims (May not Immediately Raise Suspicion)	Moderate Certifications (Local or Lesser-Known Authority)
2	Safety Gloves	Moderate Green Claims (Environmental Benefits without Proof)	Moderate Certifications (Product-Specific, Reputable Authority)
3	Copy Paper	Exaggerated Green Claims (Bold Assertions about Sustainability without Proof)	Stringent Certifications (Industry-Specific, Reputable Authority)

As prior studies indicate that both verbal and visual elements of claims and certifications significantly influence sustainable product purchases^21,25^, the experimental stimuli (product images and descriptions) were carefully designed. All product images employed herein were inspired by real-world product descriptions but were carefully adapted to anonymize brands while maintaining authenticity. A short online survey was conducted to validate the experimental stimuli.

For this purpose, 211 purchasing managers (105 in Group 1 and 106 in Group 2) were recruited through Cint—a commercial panel provider. Participants in Group 1 were exposed to two greenwashed product images and one sustainable product image, while participants in Group 2 were exposed to two sustainable product images and one greenwashed product image. This counterbalancing of product images was employed to mitigate potential bias or errors of judgment from participants validating the experimental stimuli. All participants were asked, “Please indicate the extent to which you think the product shown in this image is sustainable or greenwashed” on a five-point Likert scale (1. Genuinely Sustainable, 2. Partially Sustainable, 3. Neither Sustainable Nor Greenwashed, 4. Partially Greenwashed, 5. Fully Greenwashed). The majority of participants accurately identified the sustainability status of the products, thereby validating the stimuli’s effectiveness. This step ensured that all the images and descriptions employed herein were adequate for testing the central hypothesis^10^. This short online survey was conducted in accordance with ethical standards for social science research. No personally identifiable information was collected, and informed consent was obtained from all participants.

### Study execution

This study was conducted as part of an EU-funded research project (see Funding). A large set of data was collected through an online survey in December 2023. While a portion of the collected data has been used in previous publications under entirely different theoretical frameworks^26,27^, the data used in this study—except for participant demographics—has not been published elsewhere. To recruit participants, Cint—a commercial panel provider—was commissioned to source approximately 500 purchasing managers from EU countries, namely Belgium, France, Germany, Italy, the Netherlands, Spain, and Sweden. These countries were selected for their strong regulatory frameworks and well-established emphasis on sustainability and eco-certifications^28^.

To ensure robust data quality, a priori exclusion criteria were implemented. First, an eligibility question was used to confirm whether the participant was a purchasing manager working in an EU country. Second, two attention-check questions were embedded in the online survey to exclude disengaged participants^29^. After these exclusions, the final sample comprised 465 valid responses (232 in Group A and 233 in Group B). Most participants were well-educated and highly experienced purchasing managers from large-sized organizations (see Table 2).

**Table 2:** Participant demographics.

Characteristics	Description	Frequency		
		Group A	Group B	Combined
Age	Below 20 years	4	0	4
	21–30 years	48	53	101
	31–40 years	88	78	166
	41–50 years	53	70	123
	Above 50 years	39	32	71
Academic Qualification	High School	38	36	74
	Graduate	66	67	133
	Postgraduate	111	104	215
	Doctorate	16	22	38
	Other	1	4	5
Work Experience	Less than 2 years	21	30	51
	2–5 years	54	44	98
	5–10 years	70	78	148
	10–20 years	58	54	112
	More than 20 years	29	27	56
Country	Belgium	28	30	58
	France	29	28	57
	Germany	40	34	74
	Italy	22	30	52
	Netherlands	42	36	78
	Spain	30	36	66
	Sweden	39	37	76
	Other	2	2	4
Workplace	Micro-sized organization	26	47	73
	Small-sized organization	45	51	96
	Medium-sized organization	56	42	98
	Large-sized organization	105	93	198

As per the study framework, participants in Group A were exposed to greenwashed product images, while participants in Group B were exposed to sustainable product images. For each scenario, all participants were asked, “Compared to the price of other [laptop/safety gloves/copy paper], how much more would you be willing to pay for this [laptop/safety gloves/copy paper]?” on a nine-point Likert scale (0%, 5%, 10%, 15%, 20%, 25%, 30%, 35%, 40%) to gauge WTP differences across groups. This single-question approach to measure WTP is well-established and widely accepted in management research^30,31^.

A meta-analysis suggests that direct methods provide more accurate WTP estimates than indirect methods^32^. This study indeed used a direct approach to measure hypothetical WTP rather than an indirect approach, such as conjoint analysis. However, hypothetical WTP tends to be overestimated by approximately 20%^32^, making it necessary to apply de-biasing techniques^33^. A “cheap talk” script was included in the online survey to de-bias hypothetical WTP measurement. Hypothetical bias refers to “the deviation in a predefined aggregate or disaggregate measure due to choice data being collected in a hypothetical setting instead of a more realistic (but not necessarily naturalistic) setting”^34^. A “cheap talk” script, reminding participants to answer as if their decisions involved real financial stakes, has been widely proven to enhance response reliability^34,35^. Additionally, several other measures were taken to address potential issues inherent to online surveys or research methodology^36^. This study was conducted in accordance with ethical standards for social science research. No personally identifiable information was collected, and informed consent was obtained from all participants.

## Findings

### Scenario 1

**Fig. 1:**
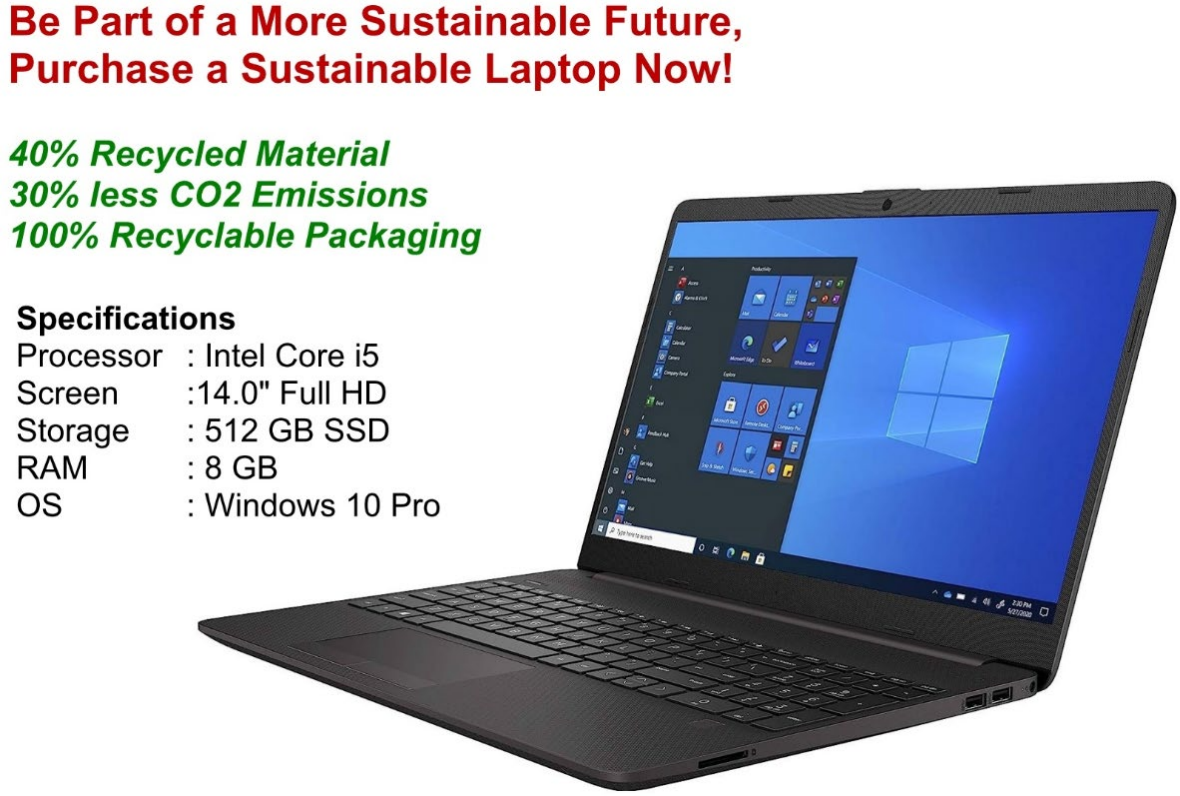
Greenwashed laptop.

**Fig. 2:**
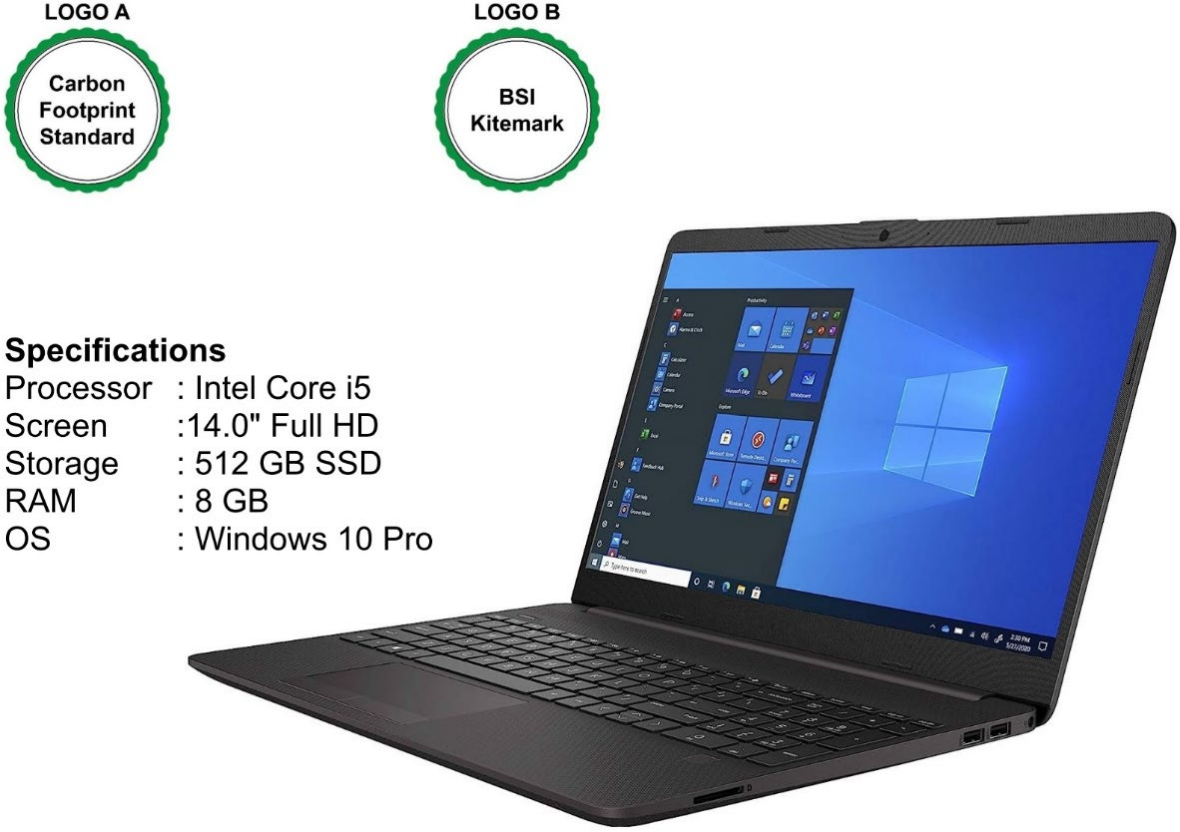
Sustainable laptop.

In Scenario 1, participants imagined purchasing a laptop for a newly recruited employee. Group A viewed a laptop with mild greenwashing claims, such as vague references to reduced energy consumption or decreased carbon emissions, unsupported by certifications (see Fig. 1). Group B viewed a similar laptop with certifications, namely the Carbon Footprint Standard – Carbon Neutral Product (Logo A) and the BSI Kitemark – Certified Remanufacturer (Logo B) (see Fig. 2). The original logos of these certifications, which were included in the experimental stimuli and shown to participants while they rated their WTP, are not displayed here to avoid any copyright issues.

**Fig. 3:**
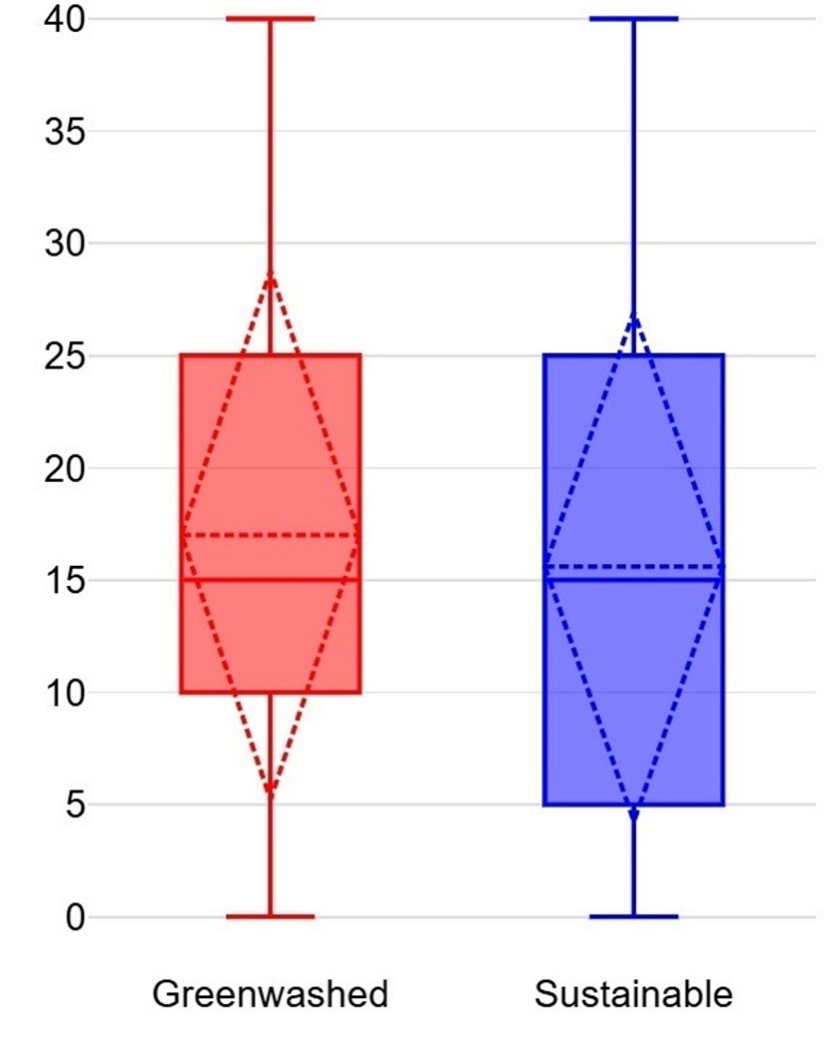
Average WTP for laptop.

An independent samples t-test (Welch’s t-test for unequal variances) was conducted to compare WTP for a laptop between Group A and Group B. The average WTP for Group A (M = 17.00%, SD = 11.80%) was slightly higher than for Group B (M = 15.60%, SD = 11.37%) (see Fig. 3), but the difference was not statistically significant, t(462) = 1.31, *p* = 0.19. These results suggest that participants did not exhibit a strong preference for a certified sustainable laptop over a greenwashed laptop. This finding implies that vague but persuasive green claims can be as influential as certifications with low perceived credibility in shaping procurement decisions.

### Scenario 2

**Fig. 4:**
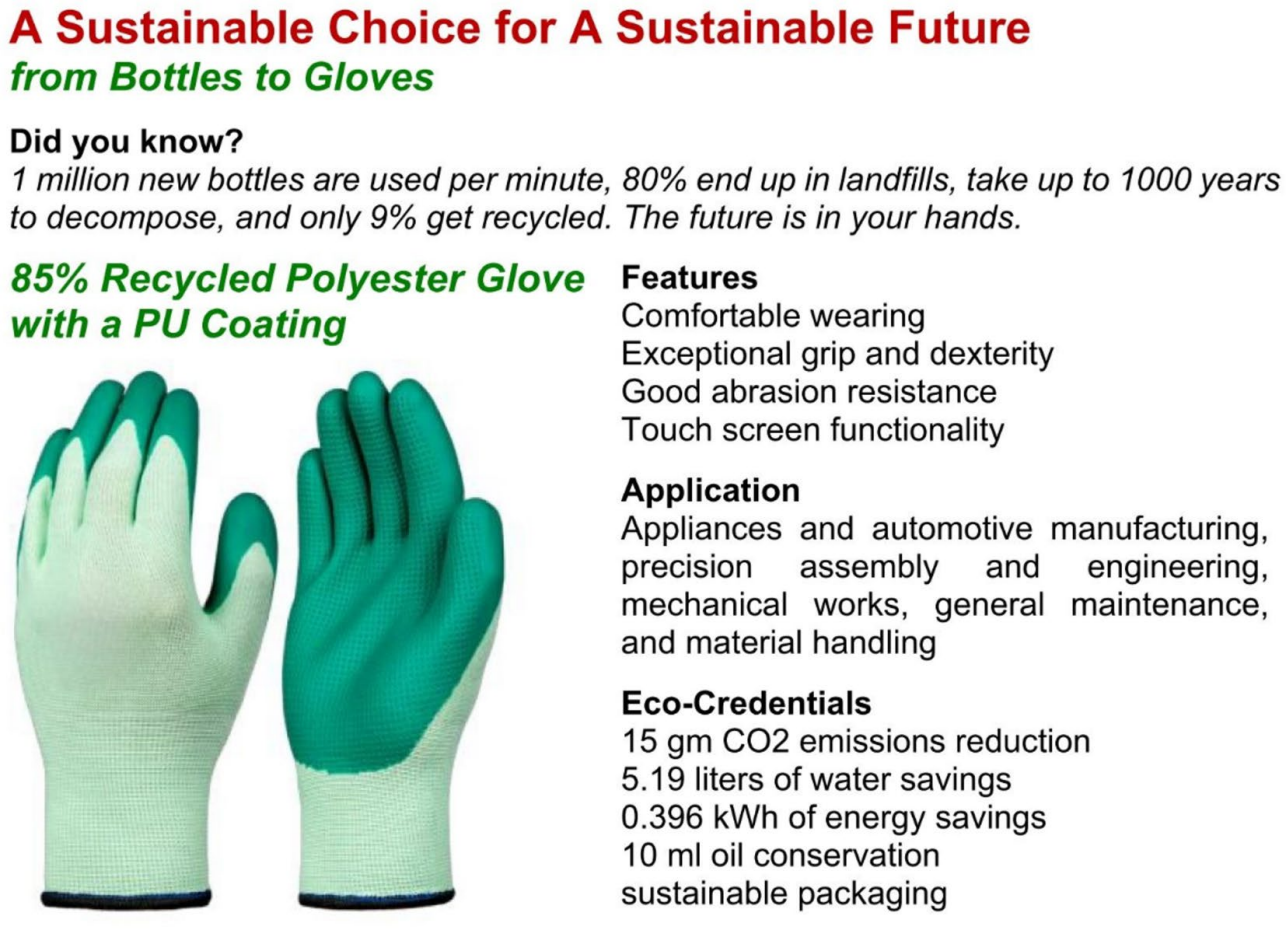
Greenwashed Safety Gloves.

**Fig. 5:**
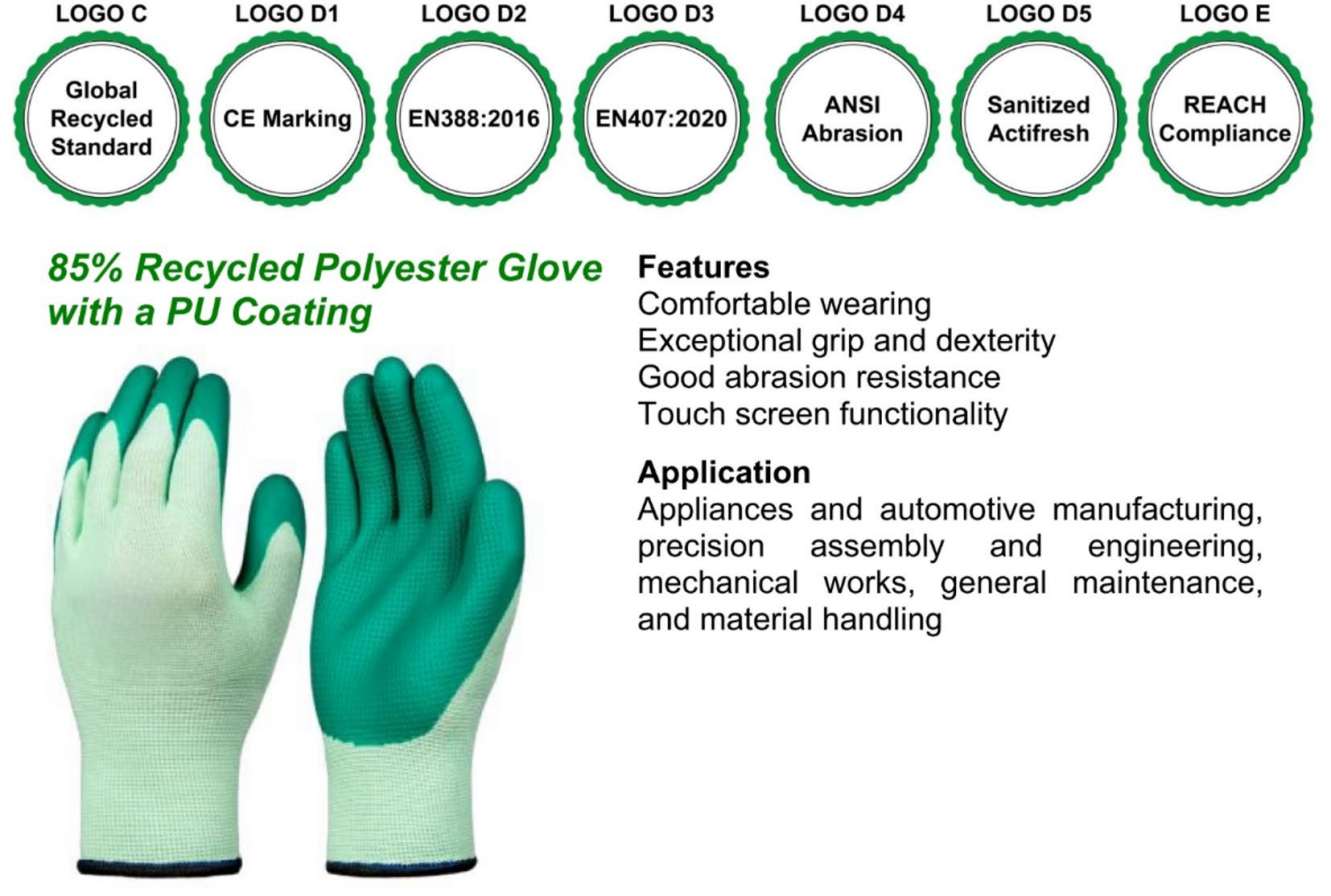
Sustainable Safety Gloves.

In Scenario 2, participants imagined purchasing personal protective equipment (PPE) for employees. Group A viewed safety gloves with moderate greenwashing claims, emphasizing environmental benefits but lacking certifications (Fig. 4). Group B viewed safety gloves with certifications or standards, namely Global Recycled Standard (Logo C), CE Marking (Logo D1), European standard for mechanical risks (Logo D2), European standard for thermal risks (Logo D3), ANSI/ISEA 105 standard for abrasion resistance (Logo D4), Sanitized Actifresh for antimicrobial protection (Logo D5), and REACH compliance (Logo E) (see Fig. 5). The original logos of these certifications or standards, which were included in the experimental stimuli and shown to participants while they rated their WTP, are not displayed here to avoid any copyright issues.

**Fig. 6:**
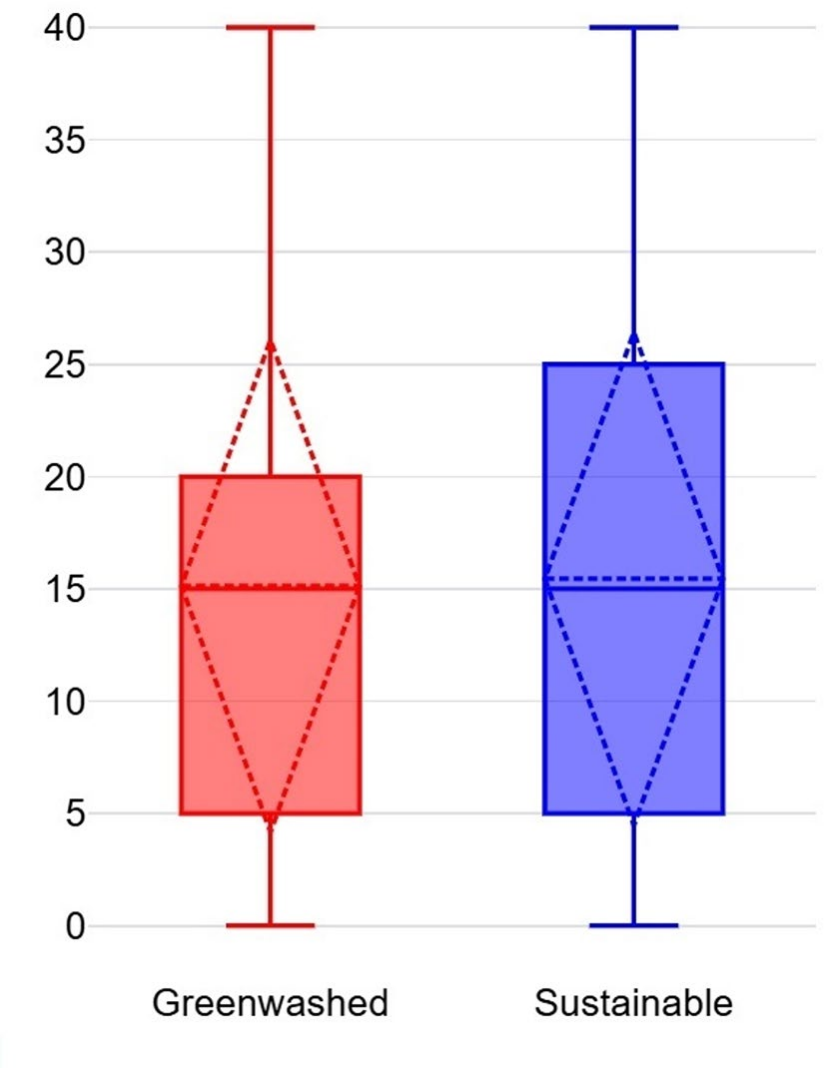
Average WTP for Safety Gloves.

An independent samples t-test (Welch’s t-test for unequal variances) was conducted to compare WTP for safety gloves between Group A and Group B. The average WTP for Group A (M = 15.13%, SD = 10.98%) was slightly lower than for Group B (M = 15.45%, SD = 11.00%) (see Fig. 6), but the difference was not statistically significant, t(463) = − 0.32, *p* = 0.75. These results suggest that participants valued certified safety gloves slightly more than greenwashed alternatives, but the difference was negligible. This finding indicates that credible certifications may carry more weight for safety–critical products, though the effect was not substantial.

### Scenario 3

**Fig. 7:**
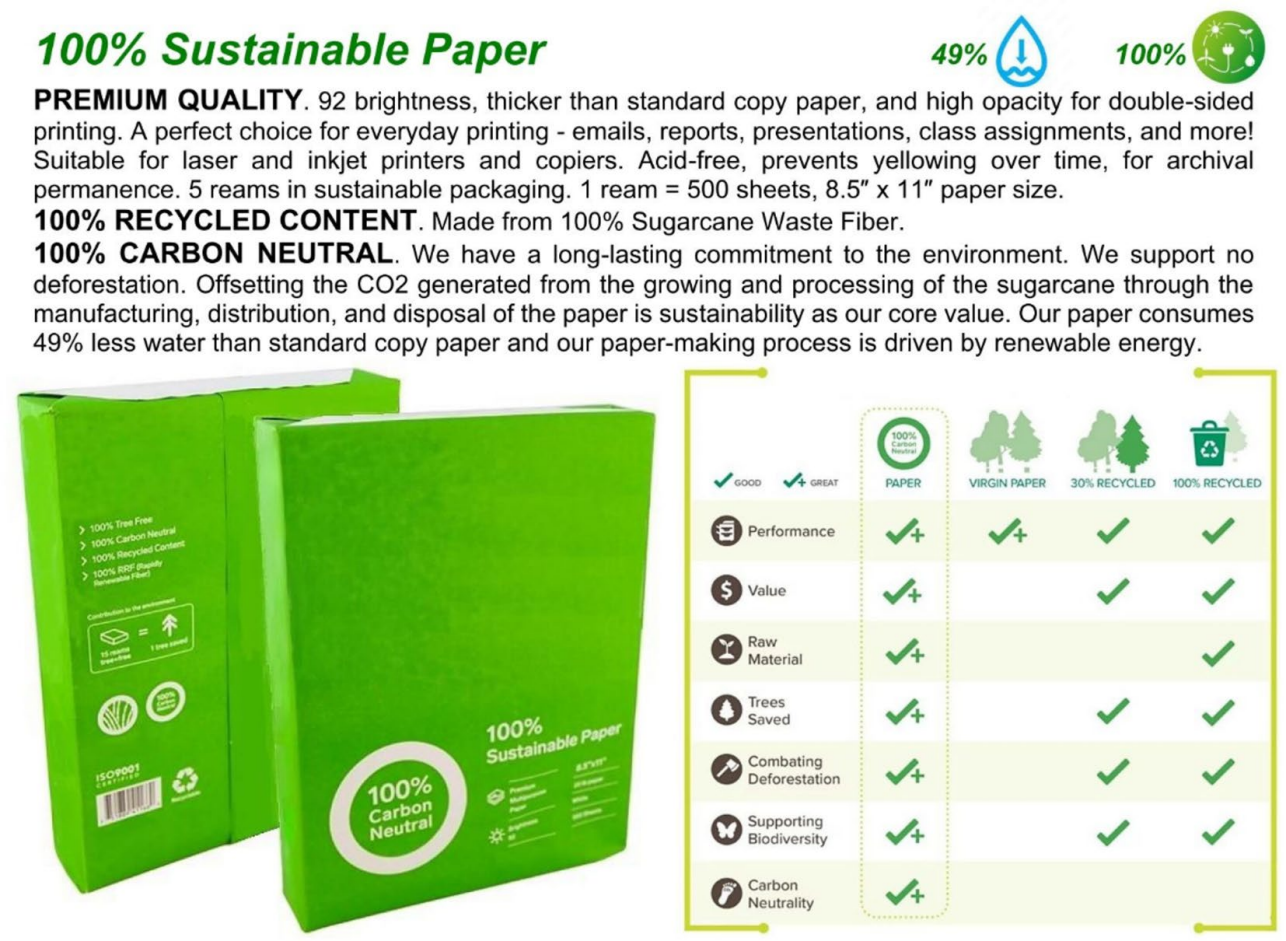
Greenwashed Copy Paper.

**Fig. 8:**
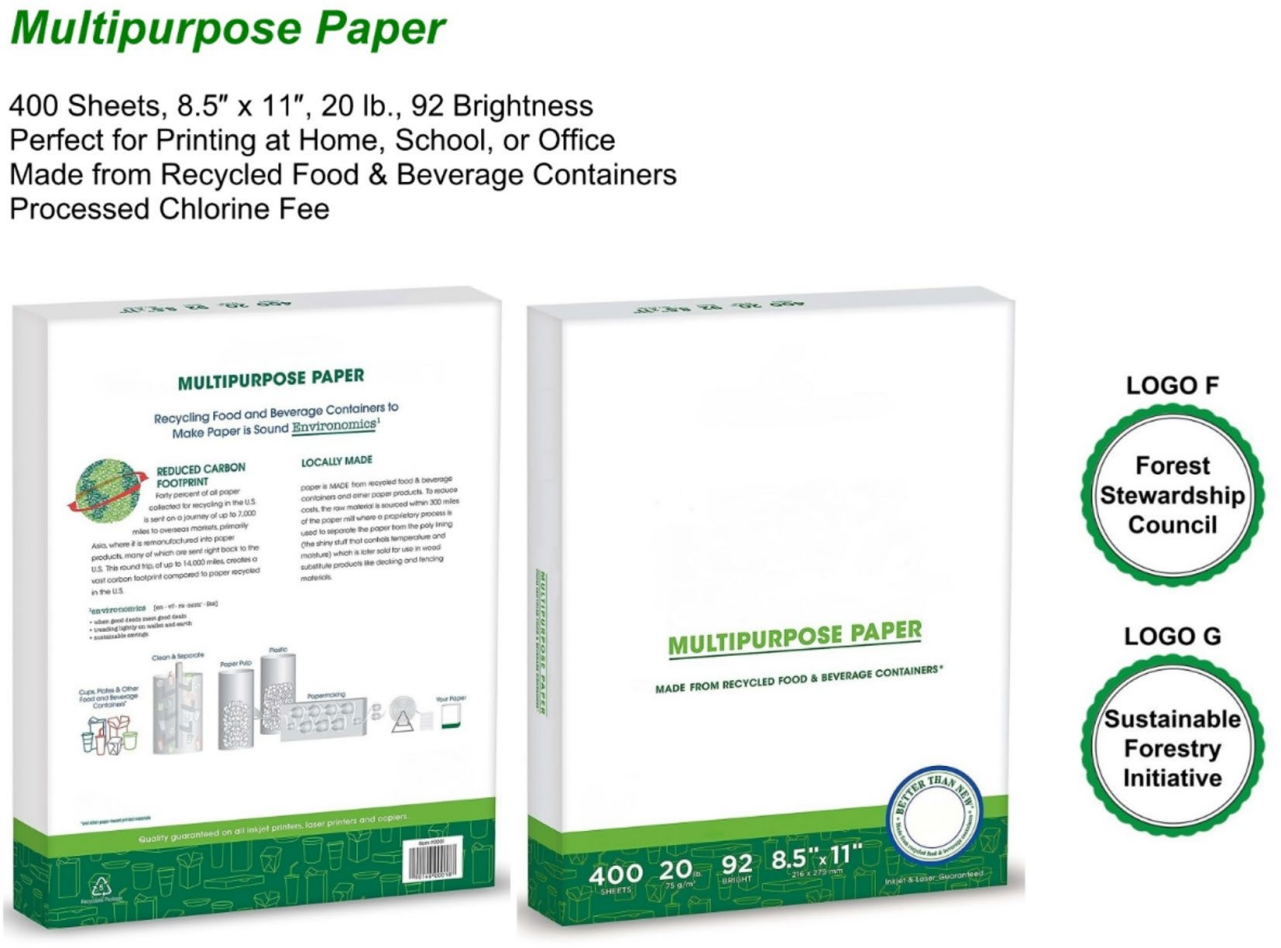
Sustainable Copy Paper.

In Scenario 3, participants imagined purchasing copy paper for their organizations. Group A viewed copy paper with exaggerated, unsupported green claims, such as “100% carbon neutral” (see Fig. 7). Group B viewed copy paper with certifications, namely Forest Stewardship Council (Logo F) and Sustainable Forestry Initiative – Certified Sourcing (Logo G) (see Fig. 8). The original logos of these certifications, which were included in the experimental stimuli and shown to participants while they rated their WTP, are not displayed here to avoid any copyright issues.

The organization used as inspiration was certified for both the Forest Stewardship Council (FSC) and the Sustainable Forestry Initiative (SFI); however, the product depicted in the experimental stimuli was certified by only one of the two, as a product can, in principle, carry only one certification at a time. In a B2B context, purchasing professionals evaluate not only the product but also the overall sustainability credentials of the supplier. They are likely to be aware of the distinction between product-level and organizational-level certifications. Given this, displaying both certifications in the experimental stimuli was a reasonable approach.

**Fig. 9:**
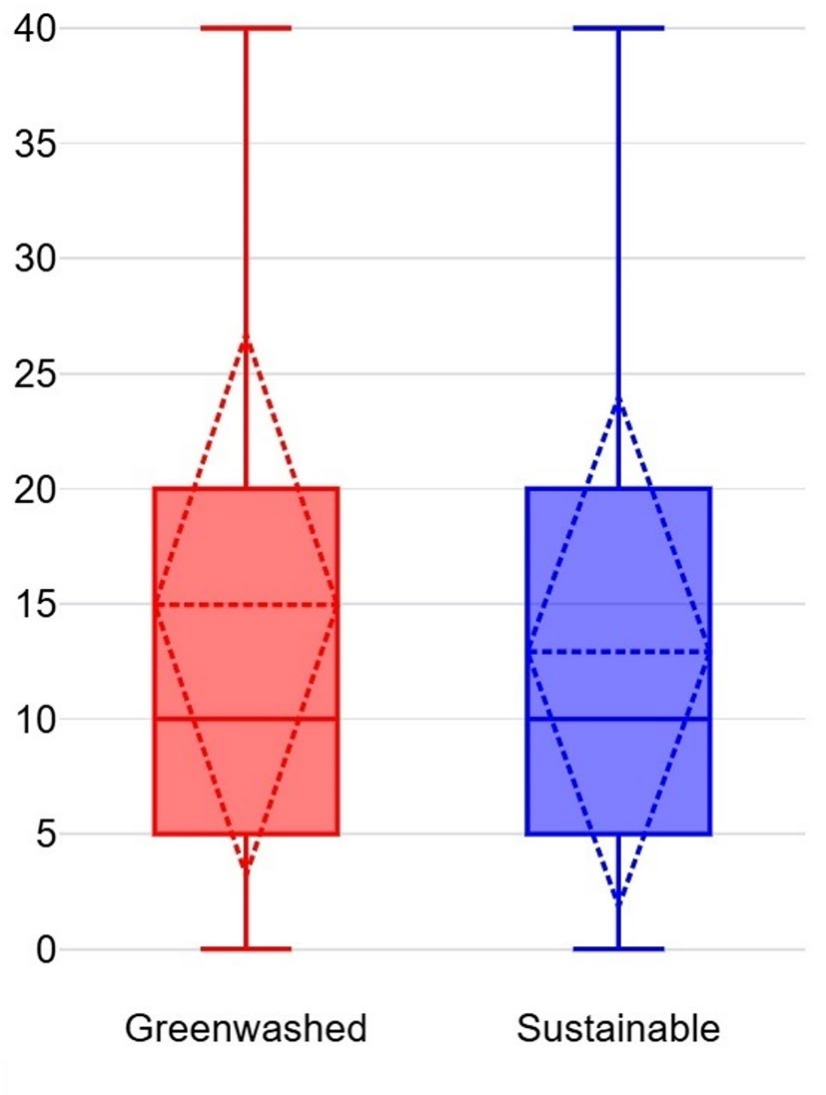
Average WTP for Copy Paper.

An independent samples t-test (Welch’s t-test for unequal variances) was conducted to compare WTP for copy paper between Group A and Group B. The average WTP for Group A (M = 14.96%, SD = 11.78%) was slightly higher than for Group B (M = 12.92%, SD = 11.09%) (see Fig. 9), but the difference was not statistically significant, t(461) = 1.92, *p* = 0.06. These results suggest that participants showed a slight preference for greenwashed copy paper over its certified counterpart. This finding highlights the influence of bold but unverifiable green claims, which can sometimes outweigh even strong certifications in procurement decisions.

## Summary

**Fig. 10:**
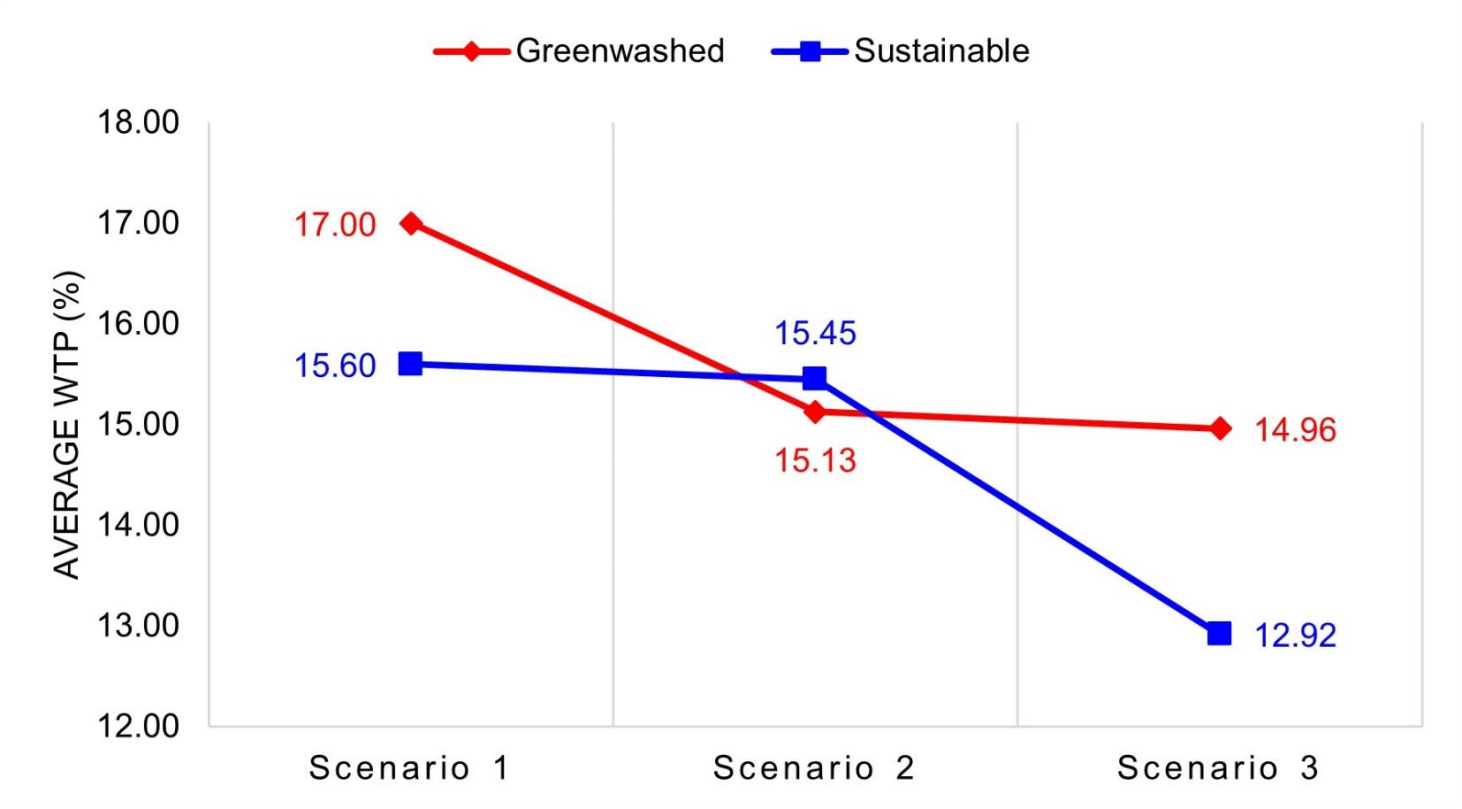
Comparison of average WTP.

The findings across all scenarios reveal varying degrees of susceptibility to greenwashing among purchasing managers. For laptops (Scenario 1) and copy paper (Scenario 3), participants exhibited a higher WTP for greenwashed products compared to certified alternatives, whereas in safety gloves (Scenario 2), participants showed a slight preference for certified products (see Fig. 10). These results indicate that purchasing managers struggle to consistently differentiate between greenwashed claims and credible certifications, particularly when persuasive yet vague environmental claims are presented.

These findings underscore a critical vulnerability to greenwashing, even among experienced purchasing professionals. This highlights the urgent need for more standardized and transparent certification systems to enhance trust in sustainability claims, support informed purchasing decisions, and mitigate the risks associated with deceptive green marketing.

## Discussion

This study highlights a critical challenge in sustainable procurement: purchasing managers, despite their expertise, are vulnerable to greenwashing. While previous research has largely focused on consumers^37,38^, the findings extend the discussion to B2B procurement professionals^39^, revealing that even experienced decision-makers fail to consistently distinguish between genuinely sustainable products and those with misleading green claims. The absence of statistically significant differences in WTP between certified sustainable products and greenwashed alternatives suggests that current certification systems and sustainability marketing practices may not be effectively guiding procurement decisions.

One of the key explanations for this finding lies in the overwhelming number of eco-labels in the market. With more than 400 different sustainability certifications worldwide^4^, the lack of standardization has created confusion not only among consumers but also among professionals^17,18^. Certifications should ideally serve as trust mechanisms that reduce information asymmetry, yet this study suggests that lesser-known certifications may fail to provide sufficient credibility, leading purchasing managers to treat them no differently from unverified green claims. This aligns with prior concerns that the proliferation of eco-labels—without clear regulatory oversight—may diminish their overall impact in guiding sustainable purchasing decisions^4^.

The findings also challenge the assumption that professional expertise shields decision-makers from cognitive biases^40^. Although purchasing managers are trained to evaluate procurement decisions critically, time constraints, organizational pressures, and overwhelming information flows limit their ability to verify sustainability claims. Behavioral decision-making theories suggest that even experienced professionals rely on heuristics when processing complex information^41^. This study demonstrates that vague but persuasive green claims were as influential as certifications, suggesting that familiar sustainability-related terms and visual cues may trigger heuristic-based decision-making rather than rational assessment of certification credibility.

Trust in sustainability claims is a decisive factor shaping procurement decisions. If certifications lack credibility due to contradictory standards, unclear messaging, or perceived industry bias, purchasing managers may default to treating all environmental claims with skepticism. This risks undermining sustainability initiatives, as professionals may struggle to differentiate between legitimate environmental efforts and marketing-driven green claims. Strengthening trust in eco-labeling requires enhanced transparency, independent verification, and harmonization of certification standards to reduce ambiguity and improve decision-making reliability^16,42^.

To address these challenges, corporations, policymakers, and regulatory bodies must take action. Organizations should integrate sustainability literacy programs for purchasing managers, ensuring they develop a critical approach to evaluating green claims. Beyond general sustainability awareness, training should focus on deceptive marketing tactics, the role of independent verification, and how to differentiate between high- and low-credibility certifications. Interactive workshops, case studies of past greenwashing scandals, and decision-making simulations could further equip procurement professionals with the tools to critically assess sustainability claims.

In addition to training, organizations should establish internal procurement guidelines that mandate a structured evaluation of sustainability claims based on third-party verification, transparency, and adherence to recognized methodologies such as life cycle assessment (LCA) and product environmental footprint (PEF)^43^. Organizations should incorporate supplier accountability frameworks, requiring vendors to provide verifiable documentation on sustainability claims. Supplier contracts could include clause-based penalties for misleading environmental claims, reinforcing the expectation of credible sustainability commitments. Blockchain-enabled verification systems could further enhance supply chain transparency, ensuring that sustainability data is both immutable and auditable^44^.

At the regulatory level, stricter standards for substantiating sustainability claims are necessary. The European Commission’s proposed Green Claims Directive aims to curb misleading environmental advertising by requiring organizations to base their green claims on scientific methodologies such as LCA and third-party audits^45^. However, to make these regulatory efforts effective, stronger enforcement mechanisms are needed. The EU’s experience with health-related advertising regulations, where only scientifically proven claims are permitted, offers a precedent for applying similar rigor to green claims. Regulatory bodies should expand market surveillance programs, conduct randomized audits of sustainability claims, and impose financial penalties for misleading greenwashing practices. Additionally, policymakers should promote real-time claim verification technologies, such as QR code-based product labeling linked to verified environmental impact data^44^.

Moreover, governments and industry associations should support the consolidation and harmonization of eco-labels, ensuring fewer, but more credible certifications. A tiered certification system, distinguishing strict, third-party verified labels from weaker, self-reported claims, could help procurement professionals assess credibility more effectively. At the same time, policymakers should incentivize organizations to adopt environmental product declarations (EPDs) and third-party verified carbon footprint disclosures, providing standardized sustainability benchmarks across industries.

This study underscores the urgent need for structural reforms in sustainability certification, regulatory oversight, and corporate sustainability education. Greenwashing has often been framed as a consumer issue, but these findings reveal that it also affects corporate procurement, with potential consequences for supply chain sustainability, regulatory compliance, and organizational credibility. Addressing this issue requires collaborative efforts from businesses, policymakers, and certifiers to enhance transparency, standardization, and trust in environmental claims.

## Conclusion

This study challenges the widely held assumption that professional expertise shields decision-makers from greenwashing. The findings reveal that purchasing managers, despite their experience, exhibit no statistically significant difference in WTP between greenwashed and certified sustainable products. This suggests that misleading environmental claims can be just as persuasive as certified sustainability credentials, raising concerns about the effectiveness of current eco-labeling systems and the ability of professionals to navigate sustainability marketing.

While this study provides valuable insights, it has certain limitations. The hypothetical scenarios used in this study, while carefully designed, do not fully replicate real-world complexities such as budget constraints, stakeholder pressures, or regulatory obligations. Future research should employ field experiments or longitudinal studies to observe actual purchasing behaviors in corporate settings.

Additionally, the study focused on generic product categories—laptops, safety gloves, and copy paper—commonly procured across industries. While this allows for broad applicability, it does not capture sector-specific procurement challenges in industries such as heavy-industry, construction, and pharmaceuticals, where sustainability considerations may be more deeply embedded in regulatory requirements. Future research should explore greenwashing vulnerability in industry-specific procurement to assess whether certain sectors are more or less susceptible to misleading green claims. Future research may also compare greenwashing susceptibility across different geographic regions, particularly in China and the U.S., where eco-labeling systems and regulatory environments differ significantly.

Another promising direction for future research is to examine individual and organizational factors that influence greenwashing susceptibility. Prior research suggests that personality traits influence green purchase behavior^46^. Future studies should investigate whether personality traits, cognitive biases, organizational culture, or firm-level sustainability commitments affect purchasing managers’ WTP for greenwashed versus certified products. Understanding these dynamics could help develop targeted interventions to reduce greenwashing vulnerability in corporate decision-making.

Greenwashing remains a pervasive challenge in sustainable procurement, affecting not just consumers, but also experienced professionals tasked with advancing corporate sustainability. This study underscores the pressing need for clearer, standardized certification systems, enhanced corporate training programs, and stronger regulatory frameworks to combat deceptive environmental marketing. Ensuring that sustainability claims drive real, rather than perceived, progress requires a collaborative effort between businesses, policymakers, and certifiers. By improving transparency, accountability, and trust in eco-labels, stakeholders can work toward a procurement landscape where sustainability decisions are based on credible, verifiable, and scientifically supported information.

## Data Availability

The data that support the findings of this study are available from the corresponding author upon reasonable request. Additionally, the survey questionnaire and the original experimental stimuli, including the certification logos used in the study, are available upon reasonable request.
